# Sepsis-induced immunothrombosis: from cellular crosstalk to multiple organ dysfunction

**DOI:** 10.3389/fimmu.2026.1919351

**Published:** 2026-09-15

**Authors:** Zhiyuan Guo, Guangyan Zhu, Min Wang, Ping Liu, Minghe Tan, Lisha Yi, Haifa Xia

**Affiliations:** 1 Department of Anesthesiology, Union Hospital, Tongji Medical College, Huazhong University of Science and Technology, Wuhan, China; 2 Institute of Anesthesia and Critical Care Medicine, Union Hospital, Tongji Medical College, Huazhong University of Science and Technology, Wuhan, China; 3 Key Laboratory of Anesthesiology and Resuscitation (Huazhong University of Science and Technology), Ministry of Education, Wuhan, China; 4 Department of Emergency and Intensive Care Unit, Tongji Hospital, Tongji Medical College, Huazhong University of Science and Technology, Wuhan, China

**Keywords:** coagulation, immunothrombosis, microvascular dysfunction, multiple organ dysfunction, sepsis

## Abstract

Sepsis is a life-threatening organ dysfunction that is caused by a dysregulated host response to infection; moreover, multiple organ failure is a major contributor to mortality. Immunothrombosis, which is driven by the coordinated activation of innate immunity and coagulation within the microvasculature, links systemic inflammation to microvascular dysfunction and organ injury. In this review, we utilize a microvascular architecture-centered framework to explain how a systemically triggered immunothrombotic response gives rise to distinct patterns of organ injury. We summarize the major mechanisms of sepsis-induced immunothrombosis, including endothelial dysfunction, platelet–neutrophil interactions, complement activation, and monocyte/macrophage-derived tissue factor expression. We further examine the differences in endothelial phenotype, barrier specialization, flow dynamics, resident immune niches, and oxygen supply–demand balance that shape injury patterns in the lungs, kidneys, heart, liver, brain, and intestines. We also discuss the diagnostic considerations and therapeutic strategies related to sepsis-induced immunothrombosis, while focusing on coagulation assessment, microcirculatory dysfunction, and the limitations of current anti-thromboinflammatory interventions. Finally, we highlight the unresolved challenge of distinguishing protective immunothrombosis from pathological thromboinflammatory injury, which may inform future efforts to improve patient stratification and risk–benefit assessments.

## 1 Introduction

Sepsis, which is defined as a life-threatening organ dysfunction caused by a dysregulated host response to infection, remains a major challenge and is a leading cause of death in critical care medicine worldwide (1). According to the latest estimates from the Global Burden of Diseases, Injuries, and Risk Factors Study (GBD), approximately 166 million sepsis cases and 21.4 million sepsis-related deaths occurred in 2021, accounting for nearly one-third of the global mortality (2). Sepsis affects multiple vital organs, including the lungs, liver, kidneys, and heart. This condition, collectively referred to as sepsis-associated organ dysfunction (SAOD), is a major cause of death in patients with sepsis (3).

The pathological course of sepsis is variable. The early phase typically features an uncontrolled hyperinflammatory response, which is frequently described as a “cytokine storm.” The latter phase may progress to prolonged immunosuppression (4). Excessive inflammatory response can further activate the coagulation system and lead to immunothrombosis. This concept was first proposed by Engelmann and Massberg (5) in 2013 and refers to an innate immune defense response that is induced by the formation of intravascular thrombi, particularly within microvessels. This process is mediated by immune cells and specific thrombosis-related molecules, which create an intravascular scaffold that facilitates pathogen recognition, containment, and destruction while protecting host integrity. During the early stages of infection, locally formed fibrin clots can trap pathogens and limit their dissemination into the systemic circulation, and this serves as an important protective mechanism (6). However, in severe sepsis, excessive coagulation activation and impaired anticoagulant mechanisms promote microthrombus formation. These microthrombi can obstruct blood flow in the microvasculature of vital organs, leading to tissue ischemia and multiple-organ dysfunction (7). Clarifying the contribution of immunothrombosis to sepsis-induced organ injury may facilitate the development of more precise interventional strategies.

From an evolutionary perspective, the coagulation system likely constitutes a component of host defense, because coagulation and immunity share homologous origins and ancient recognition/effector pathways (8). Therefore, understanding how this physiological defense mechanism becomes dysregulated and pathogenic is central to interpreting the role of immunothrombosis in sepsis. A major focus of this review is the integration of sepsis-induced immunothrombosis with organ-specific microvascular architecture. We propose that the anatomical and functional characteristics of each microvascular bed help shape the organ distribution and injury patterns of immunothrombosis. In this framework, systemic inflammation and coagulation activation provide a common upstream trigger, whereas capillary geometry, endothelial barrier specialization, oxygen supply–demand balance, local flow patterns, and resident immune cell niches shape how immunothrombotic events are translated into tissue injury. This architecture-centered perspective potentially elucidates the heterogeneous manifestations of SAOD and might inform future organ-specific therapeutic research.

This narrative review was informed by searches of the PubMed and Web of Science databases from inception to July 2026. The search terms included combinations of “sepsis,” “immunothrombosis,” “microvascular thrombosis,” “platelet,” “neutrophil extracellular traps,” “NETs,” “endothelium,” and “coagulation,” together with organ-specific terms. We prioritized original research articles, randomized controlled trials, observational studies, systematic reviews, and key mechanistic studies. The reference lists of the relevant articles were screened manually to identify additional studies. Studies were selected based on their relevance to sepsis-associated immunothrombosis, organ-specific microvascular injury, diagnostic assessments, and therapeutic strategies.

## 2 Pathophysiological mechanisms of sepsis-induced immunothrombosis

### 2.1 Structural and functional alterations in endothelial cells

Under physiological conditions, healthy endothelial cells maintain vascular patency by actively suppressing thrombosis through multiple antithrombotic mechanisms (9). However, in sepsis, pathogen-associated molecular patterns (PAMPs) and damage-associated molecular patterns (DAMPs) activate endothelial cells through pattern-recognition receptors (PRRs), including toll-like receptors (TLRs) and receptors for advanced glycation end products (RAGE) (10, 11). The resulting molecular and structural changes shift the endothelium toward proinflammatory and procoagulant phenotypes.

In sepsis, activated endothelial cells upregulate procoagulant molecules, such as tissue factor (TF), and thereby enhance local coagulation activity (12). Sepsis commonly involves reduced anticoagulant activity, including decreased levels or impaired function of tissue factor pathway inhibitor (TFPI) and antithrombin (AT), which favor microthrombus formation (9, 13). The protein C pathway is a key regulatory system that helps maintain microvascular patency by limiting excessive thrombosis and inflammatory tissue injury (14). Activated protein C (APC) reduces coagulation initiation by downregulating TF expression in monocytes via an endothelial protein C receptor (EPCR)-dependent mechanism (15). This pathway is impaired at several levels in sepsis: protein C and free protein S levels decline, whereas thrombomodulin (TM) and EPCR expression are reduced (16). Consequently, the inhibition of coagulation factors Va and VIIIa is weakened, which allows a shift toward a procoagulant, antifibrinolytic, and proinflammatory state (16). Under physiological conditions, ultra-large von Willebrand factor (UL-vWF) multimers that are synthesized and stored by endothelial cells are cleaved by ADAMTS13 to prevent excessive platelet adhesion and aggregation (17). In sepsis, ADAMTS13 activity is relatively low and its degradation may increase, leading to the pathological accumulation of UL-vWF multimers (18). These multimers bind glycoprotein Ibα (GPIbα) on platelets, and thereby promote platelet adhesion and activation on damaged endothelium and facilitate local microthrombus formation (17, 18).

Sepsis disrupts the structural integrity of the endothelium. The endothelial glycocalyx (eGC) is synthesized by the endothelial cells and covers the apical membrane. Composed mainly of heparan sulfate-rich glycosaminoglycans (GAGs) and various proteoglycans, eGC regulates vascular permeability, inhibits leukocyte and platelet adhesion, and inhibits the activation of the coagulation cascade (19). In sepsis, both the structural damage and functional loss of eGC are early features of endothelial dysfunction (20). During the early phase, many inflammatory mediators act synergistically, and sustained inflammation and oxidative stress trigger rapid degradation and shedding of eGC. Shed GAGs can form complexes with platelet factor 4 (PF4) to promote platelet adhesion to the endothelium (21). GAGs may promote thrombin generation by activating the contact system (22). Endothelial structural disruption, coupled with the acquisition of a procoagulant and antifibrinolytic phenotype, plays a central role in the pathogenesis of sepsis-induced thrombosis.

### 2.2 Platelet–neutrophil activation and crosstalk

Sepsis-induced endothelial damage promotes rapid platelet adhesion to the vascular wall, followed by platelet aggregation and amplification of immunothrombosis. Bacterial components, such as muramyl dipeptide (MDP), can activate nuclear factor κB (NF-κB) signaling in megakaryocytes, leading to upregulation of P2Y_12_, a core receptor for platelet activation (23). Upon activation, platelets release diverse chemokines that recruit neutrophils to sites of infection. This recruitment strengthens platelet–neutrophil crosstalk and accelerates microvascular thrombosis (24). This interaction is mainly mediated by the binding of P-selectin on activated platelets to the P-selectin glycoprotein ligand-1 (PSGL-1) on neutrophils (25). Furthermore, platelet P-selectin enhances coagulation by promoting TF expression in monocytes (26).

Activated platelets release mediators, such as von Willebrand factor (vWF), PF4, and thromboxane A2 (TXA2), which stimulate neutrophils to release neutrophil extracellular traps (NETs) and thereby enhance the trapping and clearance of circulating bacteria (27, 28). NETs are web-like structures that are composed of decondensed chromatin, which is decorated with granular proteins including neutrophil elastase (NE) and myeloperoxidase (MPO). NETs participate directly in antibacterial host defense (29). As NETs can extend up to approximately 50 μm, they can obstruct vascular lumens and contribute to tissue hypoperfusion and organ dysfunction (30). Moreover, NETs can induce endothelial cells to upregulate adhesion molecule and TF expression, which further promotes inflammatory cell recruitment, platelet adhesion, and amplification of the coagulation cascade (31). NETs also promote the deposition of vWF, fibrinogen, and fibrin and these provide a scaffold for thrombus formation. Histones and cell-free DNA in circulation autonomously activate coagulation independent of the normal coagulation cascade (32). Nucleosomes and histones that are released from NETs are recognized by the host as DAMPs, which then activate platelet TLR signaling, induce a procoagulant platelet phenotype, and promote thrombin generation (33). In addition, DAMPs increase cytokine release, innate immune cell recruitment, and microthrombus formation at sites of infection (34). These processes establish a positive feedback loop that sustains the coagulation activation (35). Histones in NETs can induce platelets to release polyphosphates (polyPs), which are stored in the dense granules of platelets. This process promotes the intrinsic coagulation pathway through factor XII activation, enhances factor V activation, and inhibits fibrinolysis, which collectively increases thrombin generation and activates the coagulation cascade (36–38). Moreover, thrombin activates platelets, and thereby exacerbates TF enrichment and NET formation (31).

Activated platelets release platelet-derived microparticles, which accelerate the coagulation cascade by supplying TF and negatively charged phosphatidylserine (PS) surfaces (39). Neutrophil-derived extracellular vesicles (EVs) carry substances, such as MPO and adhesion molecules, which amplify thromboinflammatory responses through platelet activation and endothelial damage (40).

### 2.3 Contribution of the complement system

In sepsis, systemic complement activation contributes to immunothrombosis by activating endothelial cells and platelets while inhibiting the anticoagulant and fibrinolytic pathways, as reviewed by Wei et al. (41). Furthermore, the complement system interacts with NETs. Complement activation is triggered by extracellular histones and nucleosomes through interactions with PRRs on immune cell surfaces (42). Conversely, the binding of C5a to its receptors C5aR and C5L2 may increase extracellular histone levels and aggravate neutrophil-dominated lung injury (43). Interactions between NETs and complement proteins amplify local inflammatory and immune responses (30). C1q, a recognition molecule of the classical complement pathway, has a positively charged spherical head that interacts with the negatively charged DNA backbone of NETs. C1q-opsonized NETs are more efficiently phagocytosed by macrophages, thereby clearing NETs and trapped pathogens, and mitigating NET-mediated tissue damage (44).

### 2.4 Role of monocytes and tissue factor

Monocytes are a major source of TF in sepsis and drive extrinsic coagulation by upregulating TF expression (45). TF binds to coagulation factor VII (FVII), and the resulting complex converts factor X (FX) to FXa in the presence of calcium ions. On phospholipid surfaces, FXa associates with Va and calcium to form a prothrombinase complex, driving thrombin generation, platelet activation, and further amplification of coagulation (46). LPS binding to TLR4 on monocytes activates intracellular signaling pathways and various transcription factors, whereas subsequent pyroptosis upregulates TF expression and promotes EV release (47). These EVs enhance the procoagulant activity of TF by promoting PS externalization to the cell surface. Additionally, they induce NET formation in neutrophils (48).

Platelet–monocyte interactions are mediated mainly through the P-selectin/PSGL-1 or CD40L/CD40 pathways. Platelet-released chemokines, including PF4 and CCL5, influence monocyte recruitment and differentiation (49). Platelets strongly promote NLRP3 inflammasome activation in monocytes (50), promote TF expression, and aggravate LPS-induced coagulation (51). In severe coronavirus disease (COVID-19) sepsis, platelets significantly upregulate the expression of TF in monocytes. Neutralizing platelet P-selectin or inhibiting platelet-monocyte aggregation by blocking integrin αIIb/β3 with the aggregation inhibitor abciximab can eliminate this platelet-mediated upregulation of TF (52). In older patients with sepsis, increased circulating platelet-monocyte aggregate (PMA) formation correlates with heightened inflammation and higher 28-day mortality (53).

These multicellular mechanisms and their reciprocal amplification are summarized in **Figure 1**.

**Figure 1:**
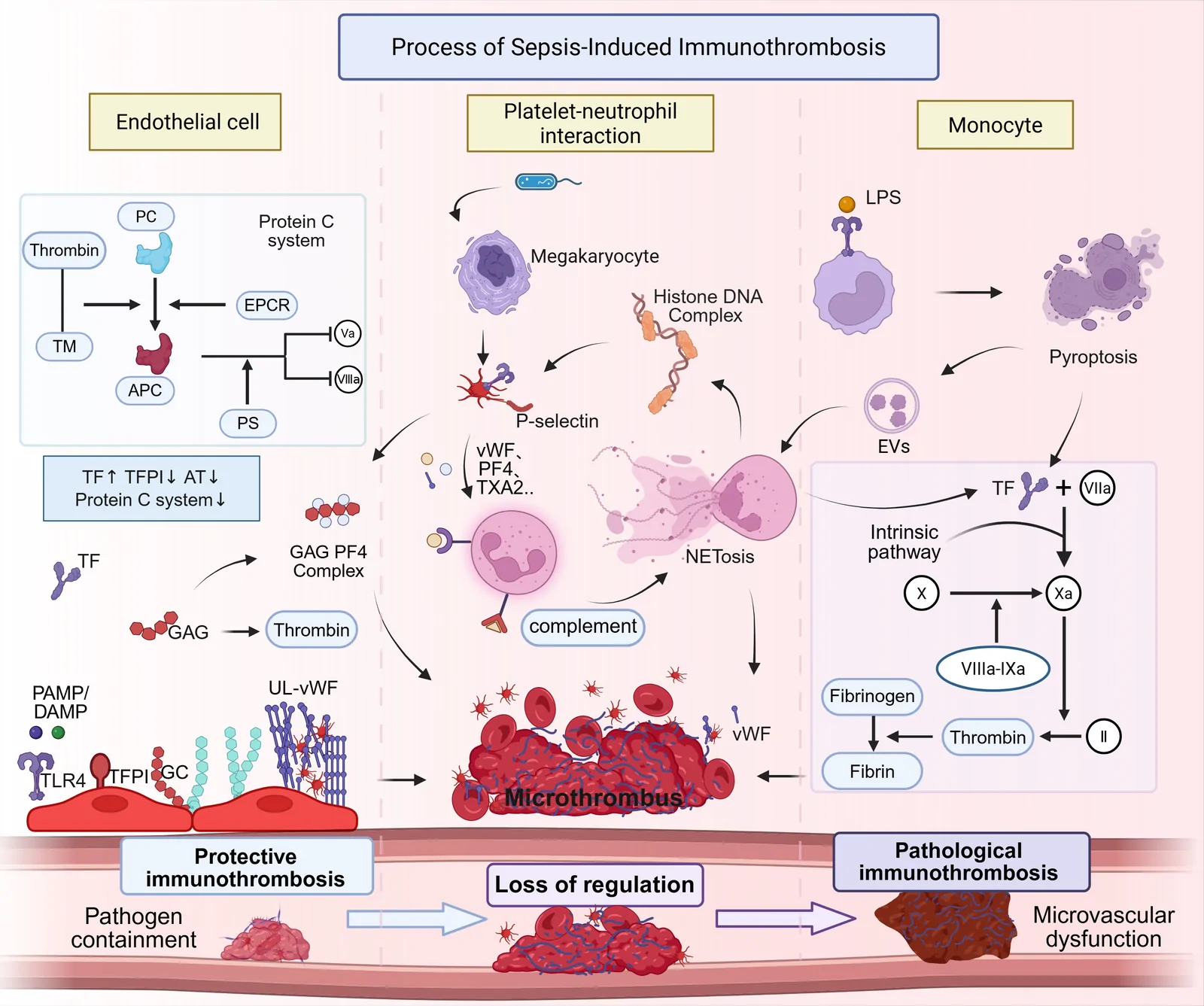
Schematic illustration of the multicellular mechanisms of sepsis-induced immunothrombosis. This schematic illustrates how coordinated responses involving endothelial cells, platelets, neutrophils, monocytes, and the complement system initially support pathogen containment but become pathological when excessive or inadequately regulated (1). Endothelial dysfunction: PAMP and DAMP recognition promotes tissue factor (TF) expression, impairs the activated protein C (APC) anticoagulant pathway, and induces endothelial glycocalyx (eGC) degradation. Shed glycosaminoglycans (GAGs) interact with platelet factor 4 (PF4), while the accumulation of ultra-large von Willebrand factor (UL-vWF) multimers promotes platelet and leukocyte adhesion (2). Platelet–neutrophil crosstalk: Activated platelets release vWF, thromboxane A2 (TXA2), and PF4, enhancing neutrophil recruitment and NET formation. NETs trap pathogens and provide a scaffold for platelet adhesion and fibrin deposition, whereas extracellular histones and cell-free DNA further amplify coagulation, complement activation, and inflammation (3). Monocyte-driven coagulation: LPS-induced monocyte activation and pyroptosis increase TF expression and the release of procoagulant extracellular vesicles, accelerating thrombin generation and fibrin formation. When anticoagulant and fibrinolytic regulation becomes insufficient, these mutually reinforcing pathways shift localized protective immunothrombosis toward widespread microvascular thrombosis, impaired tissue perfusion, and organ injury. Created in https://BioRender.com.

## 3 Platelet receptors and intracellular signaling mediators in sepsis-induced immunothrombosis

Platelet receptors and intracellular signaling mediators integrate infectious, inflammatory, vascular injury, and hemodynamic signals during sepsis-induced immunothrombosis. This section summarizes the mechanisms by which key platelet receptors and intracellular signaling pathways regulate platelet activation and the thromboinflammatory response.

### 3.1 TLR4

Among platelet toll-like receptors, TLR4 is the most extensively studied in sepsis-associated immunothrombosis (54). LPS is a canonical ligand of TLR4. LPS recognition requires the TLR4–MD-2 complex and is facilitated by accessory proteins such as LPS-binding proteins and CD14. As platelets do not express membrane-bound CD14, soluble CD14 in the plasma is required for efficient LPS sensing and complete platelet TLR4 activation *in vitro (*54, 55). Unlike nucleated immune cells, platelets lack a nucleus; thus, TLR4 signaling in platelets relies mainly on post-transcriptional and non-genomic pathways. LPS–TLR4 engagement activates MyD88-dependent signaling and the NF-κB/IKK/IκB axis, in which IKKβ promotes ATP release, thromboxane A2 generation, P-selectin exposure, platelet aggregation, and granule secretion (56). Mechanistically, IKK-mediated phosphorylation of SNAP-23 facilitates SNARE complex assembly to mediate platelet granule exocytosis (degranulation) (57). In addition, downstream pathways, such as cGMP/PKG signaling, have also been proposed to participate in the regulation of platelet granule secretion and aggregation following TLR4 activation (33, 55), although the precise signaling architecture of TLR4 in anucleate platelets remains unclear.

Platelet TLR4 activation promotes cytokine release, IL-1β synthesis, platelet–neutrophil aggregate formation, and NET formation, thereby contributing to microvascular thrombosis (58). In addition to LPS, endogenous DAMPs, such as histones, HMGB1, and heat shock protein 70 (HSP70), can interact with TLR4 and induce platelet activation (54). Thus, platelet TLR4 acts as a mechanistic bridge between infection, sterile tissue-injury signals, platelet activation, and immunothrombosis.

### 3.2 CLEC-2 and GPVI

Platelets express the immunoreceptor tyrosine-based activation motif (ITAM) and hemITAM receptors, which translate vascular injury-, inflammation-, and infection-associated signals into platelet activation. Classical platelet ITAM receptors include glycoprotein VI (GPVI) and, in humans, FcγRIIA, whereas C-type lectin-like receptor 2 (CLEC-2) contains a single YxxL motif and is therefore classified as a hemITAM receptor. Although structurally distinct, the ITAM and hemITAM receptors share several downstream signaling components (59, 60). They both converge on a shared Syk–LAT–PLCγ2 signaling axis. Ligand-induced receptor clustering triggers the phosphorylation of tyrosine residues within the ITAM or hemITAM motif, enabling Syk recruitment and activation (61). Syk subsequently phosphorylates LAT and promotes the assembly of a PLCγ2-containing signaling complex (62). PLCγ2-generated IP3 and DAG mobilize intracellular Ca²^+^ and activate protein kinase C, driving granule secretion, integrin activation, platelet aggregation, and the release of ADP and thromboxane A2. Simultaneously, PI3K-derived PIP3 recruits the Akt and the Tec-family kinases Btk and Tec to the plasma membrane, where Btk and Tec reinforce PLCγ2 signaling and platelet activation (59).

CLEC-2 is a type II transmembrane receptor of the C-type lectin-like receptor family that is highly expressed in megakaryocytes and platelets. The cytoplasmic hemITAM motif is phosphorylated upon receptor clustering, and thereby initiates Syk-dependent signaling (63). The best-characterized endogenous ligand of CLEC-2 is podoplanin (PDPN), a type I transmembrane glycoprotein that is normally segregated from circulating platelets and highly expressed in lymphatic endothelial cells (64). However, during inflammation, podoplanin is upregulated or exposed to several cell types, including macrophages, and this enables podoplanin-expressing cells to activate platelets via CLEC-2. Furthermore, CLEC-2 exerts both thrombotic and immunoregulatory effects during infection. Platelet CLEC-2 promotes hepatic thrombosis during Salmonella infection (65), whereas platelet-specific CLEC-2 deficiency or podoplanin blockade attenuates thrombotic responses in experimental settings (64). In addition to this prothrombotic function, the podoplanin–CLEC-2 axis limits systemic inflammation and organ injury in LPS- and CLP-induced sepsis (66).

GPVI is an immunoglobulin superfamily receptor that signals through its non-covalently associated Fc receptor γ-chain (FcRγ), which contains a classical ITAM with two YxxL motifs. The collagen that is exposed after vascular injury constitutes a canonical ligand for GPVI, whereas fibrin is a ligand that is capable of activating GPVI-dependent platelet signaling (60). Through FcRγ-dependent ITAM signaling and the downstream Syk–LAT–PLCγ2 pathway, GPVI links extracellular matrix exposure and fibrin formation to platelet activation. Collectively, GPVI and CLEC-2 provide platelet-specific pathways through which vascular injury, fibrin deposition, podoplanin-expressing cells, and infection-associated tissue injury amplify immunothrombosis.

### 3.3 GPIbα

The glycoprotein Ib-IX-V (GPIb-IX-V) complex is a highly glycosylated platelet-adhesion receptor that is predominantly expressed on platelets and megakaryocytes. GPIb contains the ligand-binding GPIbα subunit and GPIbβ, and associates with GPIX and GPV to form the GPIb-IX-V complex. GPIbα binds vWF, and this interaction is essential for platelet tethering and adhesion under high-shear conditions (63). Furthermore, this interaction can activate intracellular signaling pathways that involve the Src family kinases, Syk, and PLCγ2, and reinforce platelet activation (58). Beyond vWF-dependent adhesion, GPIbα participates in thromboinflammatory crosstalk with leukocytes. Mac-1 (CD11b/CD18) on leukocytes can interact with platelet GPIbα to promote monocyte TF expression through PKCδ–Foxp1 signaling and contribute to thrombus formation (67). The GPIbα–Mac-1 axis has been implicated in neutrophil activation and NET formation (63). GPIbα also contains thrombin-binding sites and may facilitate thrombin-dependent platelet activation through PAR-1 cleavage or direct GPIbα signaling (68). Thus, GPIbα links vWF-mediated platelet adhesion, leukocyte interactions, thrombin signaling, and immunothrombotic amplification, which makes it a potential therapeutic target for thromboinflammatory diseases (69, 70).

### 3.4 STING

Stimulator of interferon genes (STING) is an endoplasmic reticulum–resident innate immune signaling adaptor and a core component of the cGAS–STING signaling pathway. In nucleated immune cells, cytosolic DNA activates cGAS to generate cGAMP, which engages STING and triggers canonical TBK1–IRF3 and NF-κB signaling, leading to type I interferon responses (71). However, in anucleate platelets, STING functions through a non-canonical secretory pathway rather than classical transcription-dependent signaling. cGAMP promotes STING palmitoylation and interactions with STXBP2, facilitating SNARE complex assembly and platelet granule secretion during infection (72). Platelet-specific STING deficiency consistently reduced sepsis-induced intravascular thrombosis and NET formation in mice, which indicates that platelet STING links innate immune sensing to platelet secretion and immunothrombotic amplification (72).

### 3.5 P2Y_12_

Human platelets express two ADP-responsive P2Y receptor subtypes, P2Y_1_ and P2Y_12_, which cooperate to amplify platelet activation. P2Y_1_ couples predominantly to Gαq, whereas P2Y_12_ is classically linked to Gαi-dependent inhibition of adenylyl cyclase and potentially engages Gαq-related PLCβ signaling in platelet GPCR networks (73). Moreover, P2Y_12_ activation induces Gβγ signaling, which promotes dense-granule secretion through the PI3K/Akt pathway (73, 74). The ADP released from dense granules further reinforces P2Y_12_ signaling, and thus forms a positive feedback loop that stabilizes platelet activation and aggregation (75). Consistent with this immunomodulatory role, P2Y_12_ deficiency and clopidogrel treatment reduced platelet activation and lung injury in a murine CLP model of sepsis, whereas P2Y_1_ deficiency had no comparable effects (76).

## 4 Immunothrombosis and organ-specific injury

Sepsis-induced immunothrombosis is a central pathological mechanism that underlies systemic microcirculatory disturbances and multiple organ dysfunction. Sepsis frequently involves multiple organs, and the mortality risk increases with the number of failing organs (77). Autopsy studies of patients with sepsis demonstrated microvascular clots in numerous organs, including the kidneys, liver, lungs, intestines, adrenal glands, and brain, and the degree of organ injury correlated with the microthrombus load (78).

Importantly, the organ-specific consequences of immunothrombosis cannot be fully explained by only the activation of systemic coagulation. Each microvascular bed has distinct structural and functional properties, including endothelial phenotype, vascular architecture, barrier specialization, flow patterns, oxygen supply–demand balance, and resident immune cell composition. These properties shape where activated platelets, neutrophils, NETs, fibrin, and other thromboinflammatory mediators preferentially accumulate. Furthermore, they influence how rapidly and severely the microvascular obstruction progresses to tissue injury. Organ-specific microvascular niches transform shared systemic immunothrombotic processes into distinct organ-injury patterns. Thus, narrow alveolar capillaries favor leukocyte sequestration, whereas serial renal microcirculation and medullary hypoxia amplify perfusion deficits. In contrast, low-shear hepatic sinusoids and portal exposure to gut-derived PAMPs promote local thromboinflammation, whereas the specialized BBB makes even focal microvascular injury particularly consequential. **Table 1** summarizes these major organ-specific differences and **Figure 2** illustrates their pathological consequences.

**Table 1:** Organ-specific microvascular features and immunothrombotic injury patterns in sepsis.

Organ	Organ-specific microvascular and physiological features	Major cellular contributors to immunothrombosis	Primary thrombotic mechanisms	Typical pathological manifestations	Key evidence sources	Potential mechanistic targets or therapeutic implications
Lung	Broad, thin gas-exchange interface; dense network of narrow alveolar septal capillaries favoring leukocyte sequestration; high-capacitance, low-resistance circulation	Pulmonary microvascular endothelial cells; alveolar macrophages; platelets; recruited neutrophils	Leukocyte sequestration and mechanical obstruction; NET-rich capillary microthrombi; platelet–neutrophil interactions	Diffuse alveolar damage; hyaline membrane formation; alveolar edema, V/Q mismatch	Human autopsy studies; clinical ARDS cohorts; sepsis animal models	Alveolar barrier protection; platelet–neutrophil interactions; fibrin turnover
Kidney	Serial glomerular–peritubular circulation; high-pressure glomerular filtration; high-metabolism, oxygen-sensitive outer medulla	Glomerular and peritubular endothelial cells; platelets; recruited neutrophils and monocytes/macrophages	Endothelial activation; focal obstruction; peritubular hypoperfusion	Rare extensive necrosis; tubular injury and dysfunction; brush-border loss; peritubular fibrin deposition	Human autopsy studies; animal intravital microscopy; sepsis animal models	Endothelial and APC-pathway protection; peritubular perfusion; anticoagulant–fibrinolytic balance
Heart	Dense coronary capillary network; diastolic-dominant perfusion; high oxygen extraction and limited oxygen reserve	Coronary microvascular endothelial cells; resident cardiac macrophages; platelets; recruited neutrophils and monocytes	Focal coronary microvascular obstruction; endothelial and vasomotor dysfunction; heterogeneous microvascular perfusion	Reversible systolic and/or diastolic dysfunction; rare infarct-like necrosis; local energetic stress	Human autopsy studies; Clinical coronary flow studies; cohort coagulation markers	Coronary microcirculatory perfusion; platelet activation; endothelial–cardiomyocyte crosstalk
Liver	Discontinuous fenestrated sinusoids; low-shear portal flow with direct exposure to gut-derived PAMPs; lobular oxygen gradient; major site of coagulation factor and endogenous anticoagulant synthesis	Fenestrated, highly permeable LSECs; Kupffer cells; hepatic stellate cells; platelets; recruited neutrophils	Platelet adhesion to LSECs; sinusoidal fibrin deposition; ET-1-mediated stellate cell contraction; impaired anticoagulant synthesis	Sinusoidal hypoperfusion; cholestasis, hypoxic hepatitis; centrilobular necrosis	Sepsis animal models; human ICU autopsy studies	LSEC protection; ET-1-mediated stellate cell contraction inhibition; sinusoidal perfusion; coagulation balance
Brain	Specialized BBB and NVU; robust endothelial glycocalyx; limited collateral supply in penetrating arterioles; highly susceptible to hypoxia	Cerebral endothelial cells; pericytes; astrocytes; microglia; platelets; recruited neutrophils	NET-mediated protease injury; impaired neurovascular coupling; microglial activation; platelet- and fibrin-rich microthrombi	Focal ischemic lesions or infarction; BBB leakage; cerebral edema	Human autopsy studies; imaging and PRES evidence	BBB glycocalyx protection; thrombin–fibrin signaling; platelet activation; microcirculatory perfusion
Intestine	Physiological hypoxia at villus tips; barrier interface under intense microbial exposure	Intestinal microvascular endothelial cells; epithelial cells; mucosal macrophages; platelets; recruited neutrophils	Villus hypoperfusion and perfusion heterogeneity; tight-junction disruption; microbial translocation-driven inflammatory amplification	Intestinal barrier failure; epithelial apoptosis; bacterial and endotoxin translocation	Sepsis animal models; intestinal barrier and translocation studies	Mucosal barrier protection; microbiota-targeted therapy; gut-liver axis intervention

**Figure 2:**
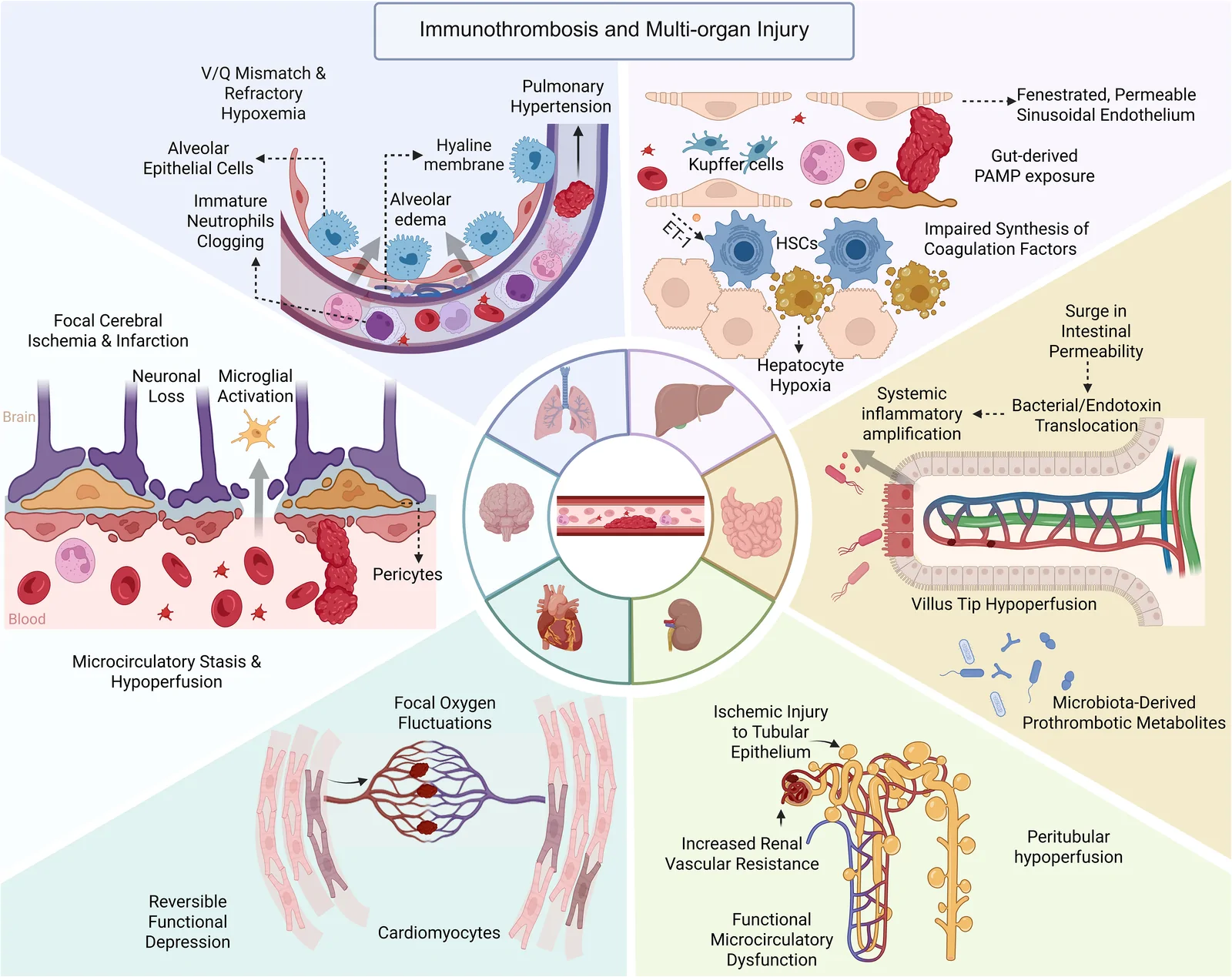
Pathophysiological mechanisms of immunothrombosis and multi-organ injury. This schematic illustrates how a shared systemic thromboinflammatory process is translated into distinct patterns of organ injury through differences in microvascular architecture, local flow, barrier specialization, immune cell composition, and oxygen supply–demand balance (1). Lung: A dense network of narrow alveolar septal capillaries favors leukocyte sequestration. Endothelial injury, platelet–neutrophil interactions, NETs, and fibrin deposition obstruct the pulmonary microcirculation, disrupt the alveolar–capillary barrier, and promote V/Q mismatch, alveolar edema, and ARDS (2). Kidney: The serial glomerular–peritubular circulation and physiological hypoxia of the outer medulla make renal oxygen delivery highly vulnerable to dynamic microvascular dysfunction. Endothelial activation, focal obstruction, and peritubular hypoperfusion impair tubular oxygenation and promote AKI (3). Heart: Despite preserved global coronary flow, focal microvascular obstruction and vasoconstriction produce perfusion heterogeneity, energetic stress, and reversible myocardial dysfunction, contributing to SICM (4). Liver: Low-shear, slow-flow hepatic sinusoidal blood is directly exposed to gut-derived PAMPs. Platelet adhesion, NET and fibrin deposition, LSEC dysfunction, and ET-1-mediated stellate cell contraction impair sinusoidal perfusion and promote hypoxic injury and cholestasis. As a major site of coagulation factor and endogenous anticoagulant synthesis, the liver can also influence systemic coagulation balance (5). Brain: Endothelial activation, glycocalyx degradation, NET-derived proteases, and microthrombi disrupt the BBB and NVU and impair neurovascular coupling, leading to BBB leakage, cerebral edema, neuronal dysfunction, and focal ischemic injury (6). Intestine: Countercurrent villus microcirculation and physiological hypoxia at villus tips increase susceptibility to hypoperfusion. Barrier disruption promotes bacterial and endotoxin translocation through the gut–liver axis, amplifying systemic inflammation and immunothrombosis. Created in https://BioRender.com.

### 4.1 Sepsis-induced immunothrombosis in acute lung injury

The lungs are particularly vulnerable to injury during sepsis (79). Sepsis-induced acute lung injury (ALI) and acute respiratory distress syndrome (ARDS) typically present with refractory hypoxemia, respiratory distress, increased alveolar–capillary permeability, and non-cardiogenic pulmonary edema (80). This susceptibility is closely related to the unique architecture of the pulmonary microvascular bed, which is optimized for gas exchange, and is highly sensitive to thromboinflammatory injury.

The pulmonary vascular system is a high-capacitance, low-resistance network with the most extensive capillary surface area in the body (81). The endothelial surface forms a continuous barrier that stringently restricts transendothelial water and protein flux, and thereby prevents interstitial and alveolar edema (82). However, during sepsis, this broad yet thin gas-exchange interface becomes a major site of endothelial activation, platelet–leukocyte adhesion, barrier disruption, and local coagulation activation. Thus, a structure that is designed to maximize diffusion can rapidly amplify the systemic thromboinflammatory signals. At the microvascular level, alveolar septal capillaries form a dense meshwork of short, interconnected segments with diameters of 5–8 μm (81). This geometry not only facilitates efficient gas exchange but also favors the retention of activated or poorly deformable leukocytes. In sepsis, immature myeloid cells, including myelocytes and metamyelocytes, exhibit reduced deformability and mechanically obstruct small vessels—an effect that is particularly evident in the pulmonary microcirculation (83). Activated neutrophils can release NETs, which further contribute to direct and widespread mechanical obstruction.

Furthermore, the pulmonary microvascular niche is distinguished by intense platelet–immune cell interactions. In the alveolar space, under homeostatic conditions, alveolar macrophages are the predominant immune cells, accounting for approximately 90% of bronchoalveolar lavage fluid cells (84). Alveolar macrophage pyroptosis exacerbates pulmonary immunothrombosis and amplifies lung injury by releasing proinflammatory and procoagulant mediators (85). This resident immune compartment explains the rapid translation of systemic inflammatory and coagulation signals into pulmonary injury. Animal studies have indicated that the pulmonary circulation serves as a major site of terminal platelet production, which contributes to approximately 50% of the total platelet output (86). This finding indicates that during sepsis, the lungs function not only as a passive target of systemic inflammation but also as a local hub for platelet production and proinflammatory signaling.

Within this structurally vulnerable and immune cell-rich pulmonary niche, retained leukocytes, NET accumulation, and platelet–leukocyte interactions provide a scaffold for pulmonary microthrombi, which promotes microvascular obstruction during sepsis. The accompanying increase in alveolar–capillary permeability impairs gas exchange, which culminates in refractory hypoxemia and ARDS (10, 87). Thrombosis or vasoconstriction can predispose regions to heterogeneous perfusion, thereby exacerbating ventilation–perfusion (V/Q) mismatch, increasing dead-space ventilation, and promoting further lung injury (88). This may explain the increased mortality rates that are associated with ARDS (89). Furthermore, microvascular thrombosis and severe injury to the microvascular bed may lead to pulmonary hypertension (PH) and acute right ventricular dysfunction, both of which result in poor clinical outcomes (89, 90). The barrier-disruptive consequences of pulmonary immunothrombosis are further reflected in the formation of fibrin-rich exudates or hyaline membranes within the alveolar spaces. This process is maintained by coagulation activation and impaired fibrinolysis, which favors the persistent formation and deposition of fibrin (91). Hyaline membranes significantly increase the diffusion distance between the alveoli and capillaries, and thereby impede oxygen diffusion (89). These pathological alterations were substantiated by postmortem findings in patients with sepsis and ARDS. Intravascular NETs composed of chromatin and MPO have been identified in the lung tissues of patients with sepsis (92). Lung tissue sections from patients with ARDS showed alveolar wall thickening, pulmonary capillary microthrombi, and interstitial edema (93).

Although sepsis induces pulmonary microthrombus formation and hypoperfusion, preclinical evidence suggests that pulmonary microthrombi can be cleared within 24 h through leukocyte-mediated fibrinolytic mechanisms (78). In animal experiments, the complete inhibition of thrombus formation potentially led to detrimental outcomes. One study demonstrated that TF deficiency resulted in alveolar hemorrhage and oxidative stress, and exacerbated ALI in mice (94); however, whether these effects reflect the coagulant or non-coagulant functions of TF remains unclear. Similarly, platelet depletion reduces survival, increases bacterial growth, and promotes pulmonary hemorrhage in pneumonia-induced sepsis (95). Reduced platelet count may impair endothelial barrier protection and facilitate pulmonary edema, which is a feature linked to ARDS progression and increased mortality (96, 97). Thrombus formation may facilitate pulmonary repair. The thrombosis-induced hypoxic microenvironment stabilizes hypoxia-inducible factors 1α and 2α (HIF-1α and HIF-2α), upregulates various angiogenic and proliferative factors, and thereby promotes the restoration of pulmonary vascular integrity following septic insult (3). However, during the acute phase of sepsis, excessive immunothrombotic injury generally outweighs the limited protective or reparative potential.

### 4.2 Sepsis-induced immunothrombosis in acute kidney injury

Sepsis-associated acute kidney injury (SA-AKI) is a common severe complication of sepsis. Its pathophysiology primarily involves microcirculatory dysfunction, cellular metabolic reprogramming, and dysregulated inflammatory responses (98). SA-AKI frequently reflects dynamic zonal microvascular dysfunction rather than widespread stable vascular occlusion.

The renal microvasculature is highly specialized and compartmentalized. The glomerular microcirculation is a high-pressure filtration interface with a relatively high shear stress (99). These hemodynamic conditions may limit stable fibrin-rich thrombus deposition. However, they do not exclude endothelial activation, leukocyte adhesion, or transient microvascular obstruction during sepsis (5). Although the kidney receives a large fraction of the cardiac output, renal oxygenation is highly heterogeneous rather than uniformly abundant. The outer medulla is particularly vulnerable because the countercurrent exchange system limits oxygen availability, with the modeled oxygen tension reaching approximately 20 mmHg (100), whereas tubular reabsorption imposes a high metabolic demand (101). In sepsis, even modest impairment of microvascular perfusion can push this region toward critical hypoxia (101). This vulnerability is further amplified by the serial organization of renal circulation. Blood flows from the glomerular capillaries through the efferent arterioles into the peritubular capillaries and the vasa recta. Thus, glomerular endothelial injury, vasoconstriction, leukocyte adhesion, or focal microvascular obstruction can reduce downstream peritubular perfusion and promote tubular hypoxia (102).

The kidney harbors one of the most abundant and diverse endothelial cell populations among all organs (103). APC mitigates endotoxin-induced AKI and reduces renal-inducible nitric oxide synthase (iNOS) expression and angiotensin II (Ang II) levels (104), which are two mediators of sepsis-associated renal hemodynamic disturbance and proximal tubular injury (105). During sepsis, impairment of the APC pathway weakens the local anti-inflammatory and anticoagulant protection (103).

Furthermore, endothelial injury and anticoagulant–fibrinolytic imbalance, including increased TF exposure, plasminogen activator inhibitor-1 (PAI-1) upregulation, and reduced endothelial nitric oxide synthase (eNOS) expression, promote microvascular thrombosis and reduce glomerular blood flow (106, 107). Glomerular capillary injury further increases microvascular permeability, favoring leukocyte stasis, microthrombus formation, and renal cortical injury (108). Microthrombi may also promote capillary shunting, diverting blood from small capillaries toward larger vessels and reducing effective renal perfusion (109). Together with endothelial leakage and leukocyte–endothelial adhesion, this microthrombus formation can increase renal vascular resistance (RVR), and further contribute to sepsis-induced AKI (110). At the tubular level, these disturbances manifest as prominent tubular injuries, including proximal tubular brush border loss, cellular necrosis, tubular luminal dilation, and cytoplasmic vacuolization, even when extensive glomerular fibrin thrombosis is not detected (111).

Autopsy studies have provided important evidence on the prevalence and distribution of renal microvascular thrombosis in patients with sepsis. An autopsy study found renal microvascular thrombosis in only 6.3% of patients with sepsis, without significant difference from noninfectious controls (112). Similarly, another autopsy study indicated that vascular thrombosis is not a major pathological feature in most cases (113). Moreover, fibrin deposition is scant within the glomeruli, whereas fibrin-stained thrombi predominantly localize to the peritubular capillaries (114). Widespread tubular necrosis is uncommon in human AKI (115), which indicates that sepsis-associated tubular dysfunction can occur without extensive necrotic injury.

This apparent discrepancy between the experimental models and human pathology should be interpreted with caution. Intravital microscopy and other high-resolution approaches used in animal studies allow real-time visualization of dynamic events, including transient platelet adhesion, leukocyte–platelet interactions, focal microemboli, and short-lived microvascular obstruction (116). In contrast, human pathological studies using autopsy or biopsy specimens provide static endpoint snapshots and may not capture the temporal evolution or redistribution of microthrombotic events. In addition, clinical interventions, including fluid resuscitation, antimicrobial therapy, organ support, and thromboprophylaxis may modify microcirculatory stasis and thrombus formation before tissue sampling. Many autopsy cohorts also included late-phase deaths, in which persistent organ dysfunction or secondary nosocomial infection may predominate over the early hyperinflammatory and prothrombotic phases that are typically modeled in experimental sepsis (117). Therefore, the low incidence of overt renal fibrin microthrombi in human autopsy studies does not exclude immunothrombotic involvement in SA-AKI but may reflect a predominantly dynamic pattern of renal microvascular dysfunction and peritubular hypoperfusion rather than widespread stable occlusive thrombosis (111).

### 4.3 Sepsis-induced immunothrombosis in myocardial injury

Sepsis-induced cardiomyopathy (SICM) is widely recognized as an acute-onset single- or biventricular systolic and/or diastolic dysfunction independent of coronary artery disease (118). Herein, SICM denotes sepsis-associated myocardial dysfunction and should be distinguished from sepsis-induced coagulopathy (SIC). In the heart, sepsis-induced immunothrombosis manifests less as infarction-like necrosis and more as microvascular perfusion heterogeneity, energy mismatch, and reversible myocardial dysfunction.

This injury pattern is closely related to the functional organization of the coronary microcirculation. The myocardium has an exceptionally dense capillary network and a high resting-oxygen-extraction rate of 70%–80%, which leaves a limited oxygen reserve and makes oxygen delivery highly dependent on coronary blood flow (119). Myocardial perfusion primarily occurs during diastole and creates a phasic perfusion pattern. These phasic reductions in the myocardial microvascular flow may provide temporal windows for the adhesion and retention of thrombotic components (120, 121). Early animal studies suggested that SICM occurs secondary to global myocardial ischemia owing to insufficient coronary blood flow. However, this hypothesis was challenged by the clinical findings of Cunnion et al., who demonstrated that coronary blood flow remained normal or even increased in patients with septic shock and concurrent myocardial dysfunction (122). Furthermore, studies in septic animal models showed no evidence of myocardial hypoxia and the substrate delivery to the myocardium remained uncompromised (123). Comparative analysis using thromboelastography suggested that microvascular thrombosis is not the primary mechanism for elevated cardiac troponin I (cTnI) in patients with severe sepsis (124).

Collectively, these findings indicate that large-vessel or global coronary blood flow insufficiency is unlikely to be a primary driver of SICM, although microvascular perfusion heterogeneity remains an important factor (123). Given the exceptionally high metabolic demand and limited cardiac oxygen reserves, even transient focal microthrombi may create patchy microvascular perfusion defects, which lead to transient oxygen fluctuations, local energy stress, and cardiomyocyte dysfunction. Macrocirculatory and microcirculatory hemodynamics are closely linked. Impaired coronary microcirculatory flow can trigger metabolic shutdown in underperfused areas, and thereby contribute to functional and energetic abnormalities in the septic heart (125). As cardiomyocytes and capillaries are in close proximity, local perfusion heterogeneity, endothelial activation, and inflammatory mediators can rapidly alter cardiomyocyte energy status (121). Restoration of flow after microcirculatory obstruction may precipitate ischemia–reperfusion injury, which is marked by bursts of reactive oxygen species (ROS), calcium overload, amplified inflammation, and additional cardiomyocyte damage (126).

In patients with septic shock, the index of microcirculatory resistance (IMR) was significantly elevated, which supports the presence of coronary microvascular dysfunction (127). Moreover, coagulation activation seems clinically relevant. In a prospective cohort of 908 patients with sepsis, coagulation-related markers, including platelet count, D-dimer, protein C, and AT, independently predicted elevated cardiac troponin levels (128).

Autopsy evidence supports this functional injury pattern. Widespread cardiomyocyte death is rare in SICM and substantial microthrombi are predominantly confined to small coronary vessels (118). Thus, septic myocardial injury is better understood as a reversible disorder involving microvascular perfusion, endothelial–cardiomyocyte communication, mitochondrial function, and contractile regulation, rather than irreversible necrosis caused by global coronary obstruction.

### 4.4 Sepsis-induced immunothrombosis in hepatic injury

The liver is an essential metabolic and immune organ. Sepsis-induced liver injury (SILI) may manifest as hypoxic hepatitis, cholestasis, or hepatocellular injury that is driven by excessive inflammation (129). In the liver, systemic immunothrombosis is translated by the distinctive architecture of the hepatic sinusoid, abundant resident macrophages, and a marked oxygen gradient across the hepatic lobule.

Sinusoidal liver endothelial cells (LSECs) differ substantially from continuous vascular endothelial cells. They lack a continuous basement membrane and contain open fenestrae that create a discontinuous and highly permeable exchange interface (130). This structure facilitates the exchange of solutes and macromolecules while also supporting immune surveillance and pathogen filtration. However, during sepsis, this open architecture exposes the sinusoidal niche to circulating inflammatory mediators and gut-derived PAMPs delivered through the portal system (131). Consequently, LSECs, Kupffer cells, platelets, and recruited neutrophils are activated, creating a permissive local environment for thromboinflammatory amplification. LSEC dysfunction promotes sinusoidal hypoperfusion, and inflammatory signaling induces iNOS expression in LSECs, contributing to sinusoidal endothelial dysfunction, hepatocellular cholestasis, and liver injury (129, 132). LSECs also secrete endothelin-1 (ET-1), which acts on hepatic stellate cells (HSCs) to induce their contraction, compressing hepatic sinusoids, and further reducing blood flow (133). Although emerging evidence suggests that the interactions between LSECs and platelets are involved in the regulation of hepatocyte proliferation (134), excessive platelet adhesion may exacerbate sinusoidal stasis and immunothrombotic injury.

The hemodynamic properties of the hepatic sinusoids further shape this response. Portal venous blood enters a honeycomb-like sinusoidal network with slow, low-shear flow. This architecture facilitates pathogen detection and immune cell capture under physiological conditions; however, during sepsis, it can favor platelet adhesion to LSECs, leukocyte recruitment, and local retention of thromboinflammatory mediators (135, 136). In septic rats, LPS exposure reduces thrombomodulin expression in LSECs, alters vWF release and distribution, and induces fibrin deposition within hepatic sinusoids, accompanied by hepatocellular degeneration and focal necrosis (137).

The liver contains one of the largest populations of macrophages in the body. In early sepsis, platelets cooperate with Kupffer cells to promote bacterial clearance before other innate immune responses are activated (138). However, excessive Kupffer cell activation results in the release of inflammatory and procoagulant mediators that amplify local thromboinflammation. Neutrophils undergo NETosis, which promotes sinusoidal microthrombus formation, compromises sinusoidal perfusion, and contributes to hepatocyte injury (139). These changes exacerbate tissue damage, recruit neutrophils and establish a vicious cycle of hepatic thromboinflammation. The resulting sustained thromboinflammatory milieu may enhance extracellular matrix deposition and promote hepatic fibrogenesis (140, 141). Moreover, the physiological oxygen gradient from the periportal to pericentral region renders the pericentral zone particularly vulnerable to sinusoidal hypoperfusion and microthrombus-induced hypoxic hepatocellular injury (142). Because the centrilobular region has the lowest baseline oxygen tension, sinusoidal microthrombi preferentially promote pericentral necrosis. Centrilobular necrosis around the central vein was consistently observed in 12 of 15 ICU patients who died of sepsis (143).

The liver is the primary site of synthesis of most coagulation factors. SILI impairs hepatic synthetic capacity, leading to reduced production of coagulation factors and further disturbance of the coagulation balance (144). In addition, decreased synthesis of hepatically derived anticoagulants, including AT-III and protein C, compromises the anticoagulant system. Reduced AT-III levels and elevated fibrin degradation product (FDP) levels reflect coagulation dysfunction and are important predictors of SILI (145). AT-III may inhibit microthrombosis and promote hepatocyte regeneration, while FDPs may influence sepsis-associated liver injury by modulating coagulation function (145).

Collectively, the distinct sinusoidal architecture, low-shear hemodynamics, abundant resident immune niche, and zonal oxygen gradient of the liver jointly shape its immunothrombotic phenotype. Sepsis-induced hepatic injury is closely associated with sinusoidal microthrombosis and hypoperfusion, and this is driven by the combined effects of local thromboinflammatory amplification, systemic coagulation disturbance, and impaired hepatic synthetic function.

### 4.5 Sepsis-induced immunothrombosis in cerebral injury

Sepsis-associated encephalopathy (SAE) is a type of cerebral dysfunction that occurs secondary to sepsis. Its pathophysiology involves blood–brain barrier (BBB) disruption, neuroinflammation, mitochondrial dysfunction, neurotransmitter imbalance, and microcirculatory dysregulation (146). In the brain, sepsis-induced immunothrombosis can produce focal ischemic lesions through small-vessel thrombosis and simultaneously disrupt the BBB and neurovascular unit (NVU) to promote neuroinflammation and impair neurovascular coupling.

The BBB is a highly specialized neurovascular interface between the peripheral circulation and brain parenchyma. Its integrity is essential for the maintenance of cerebral homeostasis and physiological neuronal function (146, 147). Tight junctions supported by pericytes and astrocytes, together with low expression of adhesion molecules on endothelial cells, restrict neutrophil extravasation (148). The eGCs in the brain form a more formidable barrier than those in other organs, and exhibit the greatest luminal surface coverage and thickness among systemic capillaries (149). Beyond its barrier function, the brain microvascular network is a core component of the NVU that matches local blood flow to neuronal activity (150). Therefore, immunothrombotic injury may disrupt neurovascular coupling by damaging multiple NVU components (151), which is an important feature that distinguishes brain injury from injury in other organs.

During sepsis, endotoxemia and systemic proinflammatory cytokines activate cerebral endothelial cells (152). Circulating enzymes, such as heparanase and elastase, degrade cerebral eGC, expose adhesion molecules, and facilitate platelet and leukocyte retention within the cerebral microvasculature (153). Activated platelets may release IL-1β whereas NET-derived neutrophil elastase and MMP-9 can degrade tight-junction proteins, such as occludin and claudin-5. These processes weaken the endothelial tight junctions and increase BBB permeability. Following BBB disruption, plasma fibrinogen extravasates into the brain parenchyma where it activates microglia, enhances their phagocytic activity, and exacerbates SAE pathology and barrier dysfunction (154). Activated microglia are frequently located close to endothelial cells, which suggests a link between microglial activation and endothelial dysfunction (155). Thrombin converts soluble fibrinogen to fibrin, activates platelets, sustains microthrombus formation, aggravates focal ischemia, and increases BBB permeability (152). BBB disruption leads to cerebral edema and diminished microvascular perfusion, which exacerbates neuronal loss in SAE (152). Microthrombi and inflammatory mediators may induce pericyte contraction or apoptosis, potentially disrupting local microcirculatory regulation.

The brain is highly susceptible to these pathological alterations, because cerebral function depends on continuous oxygen and glucose delivery. The limited collateralization of deep-penetrating arterioles and high susceptibility to hypoperfusion render the brain vulnerable to rapid neuronal energy failure following microembolism or regional hypoperfusion (120). Autopsy neuropathological studies provided evidence of cerebral ischemic injury in sepsis. In patients with septic shock, the hypercoagulability syndrome was identified in 9% of cases and was characterized by the presence of fibrin microthrombi in multiple small intracerebral blood vessels (156). Another autopsy revealed extensive cerebral infarction caused by neutrophil- or fibrin-mediated small-artery occlusion. These findings indicate that sepsis-associated coagulation activation and microvascular thrombosis can lead to focal ischemia and cerebral infarction (157). A retrospective propensity score-matched cohort study found that aspirin use was associated with improved short- and long-term survival in patients with SAE without a significant increase in gastrointestinal bleeding or thrombocytopenia (158).

In addition, the radiological features that are observed in sepsis may overlap with those seen in endothelial activation–related disorders, such as thrombotic thrombocytopenic purpura (TTP) (159). TTP is a known cause of posterior reversible encephalopathy syndrome (PRES), and the radiological features of PRES involve extensive vasogenic edema of the parietal and occipital lobes. These pathological changes occur secondary to endothelial cell activation, microthrombosis, and the subsequent disruption of the BBB. In a cohort of 106 patients with PRES, 23 cases were attributable to sepsis; magnetic resonance angiography (MRA) that was performed on a subset of these patients revealed microcirculatory stasis in the brain (160).

These findings suggest that brain immunothrombosis manifests as a combined pattern of focal microvascular ischemia and diffuse BBB/NVU dysfunction, which distinguishes SAE from injury patterns that are driven primarily by perfusion failure or barrier disruption.

### 4.6 Sepsis-induced immunothrombosis in intestinal injury

The intestine constitutes the largest immune barrier in the human body and protects the host from intestinal microbes and toxins. Sepsis severely disturbs the composition of the intestinal microbiota and the resulting dysbiosis further impairs intestinal barrier function and local immunity (161). In the intestine, sepsis-induced immunothrombosis leads to barrier failure via villus microcirculatory dysfunction, epithelial hypoxia, endothelial activation, and microbial translocation.

Intestinal barrier dysfunction is a common complication of sepsis. Visceral hypoperfusion and intestinal microcirculatory dysfunction are critical early events that lead to intestinal injury and barrier disruption (162, 163). The intestinal villi possess a unique microanatomy in which the arterioles and venules run in parallel, creating a steep oxygen gradient from the base to the apex (164). This countercurrent-exchange mechanism maintains physiological hypoxia at the tip of the villus under homeostatic conditions (164). Although the intestinal epithelium adapts to chronic physiological hypoxia via HIF signaling, this adaptation does not enhance its tolerance to ischemic injury (165). In sepsis, physiologically hypoxic villus tips experience marked perfusion impairment, leading to intestinal barrier dysfunction. Intestinal barrier disruption involves the downregulation of tight junction proteins, including claudins, occludin, and zonula occludens-1, thinning of the mucus layer, increased epithelial apoptosis, and consequent increase in intestinal permeability (166). Intestinal barrier dysfunction can be viewed as both a consequence and perpetuating cause of sepsis (167). Once this barrier is compromised, bacteria and their byproducts gain rapid access to the liver and systemic circulation through the portal venous system, which exacerbates the inflammatory response and contributes to multiorgan injury (167, 168).

In addition to the inflammatory consequences of intestinal barrier dysfunction, gut microbiota-derived metabolites such as trimethylamine N-oxide (TMAO) may promote thrombosis (169). Therefore, strategies that preserve intestinal microcirculation and reinforce the mucosal barrier may help interrupt the gut-derived amplification of systemic inflammation and immunothrombosis.

## 5 Diagnostic assessment and therapeutic strategies for sepsis-induced immunothrombosis

### 5.1 Diagnostic assessment of pathological immunothrombosis

The dual role of immunothrombosis in host defense and organ injury complicates therapeutic decision making. The transition from protective to pathological immunothrombosis may unfold during a dynamic window rather than at a discrete time point. This window is characterized by persistent coagulation activation, endothelial injury, microcirculatory impairment, and progressive organ dysfunction. Currently, no biomarker or diagnostic modality can reliably identify this transition. Clinical scoring systems such as the sepsis-induced coagulopathy (SIC) score proposed by the International Society on Thrombosis and Haemostasis (ISTH) Scientific and Standardization Committee provide a practical framework for assessing sepsis-associated coagulopathy (170). Dynamic changes in platelet count, antithrombin, protein C, protein S, activated partial thromboplastin time (APTT), D-dimer, fibrinogen, PAI-1, and thrombomodulin may reflect the severity of the host response and have prognostic significance (171–173). Viscoelastic tests, including rotational thromboelastometry (ROTEM), thromboelastography (TEG), and spatial clot-growth assays, can further characterize whole-blood coagulation dynamics, fibrinolytic resistance, hypercoagulable states, and thrombotic risk in patients (174–176).

Pathological immunothrombosis causes organ injury partly through microvascular dysfunction. Accordingly, coagulation biomarkers should be interpreted along with the microcirculatory assessment. Sublingual microcirculation imaging using sidestream darkfield or incident dark-field techniques allows bedside visualization of microvascular perfusion, and the perfused boundary region may provide indirect information on eGC integrity (20). Other approaches, including laser Doppler flowmetry, laser speckle contrast imaging, handheld microscopy, and intravital microscopy, can assess microvascular perfusion and leukocyte–endothelial interactions, whereas electron microscopy can characterize ultrastructural endothelial and glycocalyx injury (177–180). Thus, future therapeutic strategies may be better guided by a dynamic multimodal assessment integrating coagulation biomarkers, microcirculatory imaging, endothelial injury markers, and organ-dysfunction trajectories, rather than by any single biomarker threshold. However, no validated cutoffs or composite criteria are currently available, and the clinical utility of such an approach requires prospective validation.

### 5.2 Infection control and supportive care as the therapeutic foundation

Sepsis management requires early recognition, timely antimicrobial therapy, source control, organ support, and prevention of secondary complications. As infection is the initial trigger of sepsis, current management emphasizes prompt empirical antimicrobial therapy, particularly in patients with septic shock, followed by pathogen-directed de-escalation once microbiological and susceptibility data are available (181). Adequate source control, including the drainage of abscesses, repair of perforated viscera, and removal of infected devices, is essential for improving outcomes (181). By reducing the microbial burden and exposure to pathogen-associated molecular patterns, effective anti-infective therapy provides an essential basis for limiting subsequent thromboinflammatory responses.

Beyond these foundational measures, increasing attention has been directed toward therapeutic strategies that specifically target sepsis-associated coagulopathy and immunothrombosis. Pharmacological thromboprophylaxis is also recommended for the prevention of venous thromboembolism (VTE) when not contraindicated, although it still carries the risk of bleeding. These interventions should be distinguished from the standard anti-infective therapies. Their potential benefits must be carefully balanced against bleeding risk and interference with host defense. Although numerous anticoagulants have been evaluated in clinical trials, no anticoagulant strategy has achieved broad international guideline endorsement as a specific therapy for sepsis-associated DIC (182).

### 5.3 Host defense peptides/antimicrobial peptides as immunothrombosis-modulating agents

Host defense peptides (HDPs), also known as antimicrobial peptides (AMPs), are small cationic peptides that are produced by immune and epithelial cells. Major human HDPs include the cathelicidin LL-37 and α/β-defensins. Although traditionally regarded as broad-spectrum antimicrobial molecules, HDPs are increasingly being recognized as pleiotropic regulators of innate immunity, platelet activation, coagulation, and thromboinflammation (183, 184). Platelets act as reservoirs and effector cells for HDPs and release these peptides upon activation. Oligomerized LL-37 can engage platelet GPVI, activate the Syk–PLCγ2 pathway, lower the threshold for platelet activation, and promote platelet–neutrophil aggregate formation and NET release, thereby amplifying thromboinflammatory responses (183, 185).

Therapeutically, HDPs are promising but challenging. Recent preclinical studies of Cath-MH, Cath-HG, and Cath-LW have demonstrated that selected cathelicidins can improve outcomes in experimental sepsis through distinct mechanisms, including antimicrobial, LPS-neutralizing, antiplatelet, and anticoagulant effects (186–188). Notably, Cath-HG inhibits GPVI-mediated platelet activation, underscoring the peptide-specific effects of cathelicidins on thromboinflammation (188). These findings support the potential of HDP/AMP-based strategies to combine antimicrobial activity with the modulation of inflammation, platelet activation, and coagulation. However, excessive HDP-induced platelet activation may aggravate thrombosis, platelet consumption, and NET-mediated tissue injury (189). The therapeutic window for preserving antimicrobial defense while limiting thromboinflammation remains undefined. Natural LL-37-based interventions are limited by rapid proteolytic degradation, pharmacokinetic uncertainty, and potential off-target cytotoxicity.

### 5.4 Additional targeted anti-thromboinflammatory strategies

In addition to HDP/AMP-related approaches, several targeted strategies have been explored to modulate the excessive thromboinflammatory responses in sepsis. Among them, anticoagulant therapy has received the most attention. Heparin has been extensively studied because of its anticoagulant and anti-inflammatory properties (190). However, whether heparin improves long-term outcomes of sepsis remains uncertain, and the clinical relevance of its anti-inflammatory effects requires further evaluation. P2Y_12_ receptor is critical for platelet aggregation and thrombosis. In human experimental endotoxemia, P2Y_12_ inhibition reduced platelet–monocyte aggregate formation and the peak concentrations of several proinflammatory cytokines, supporting both antiplatelet and anti-inflammatory effects (191, 192).

The APC system exerts its classical anticoagulant effect by proteolytically inactivating coagulation factors Va and VIIIa (14). In the PROWESS trial, drotrecogin alfa (activated) (DrotAA), which is a recombinant form of human APC, significantly reduced 28-day all-cause mortality in patients with severe sepsis (14). However, subsequent trials, including ADDRESS and PROWESS-SHOCK, failed to confirm a consistent survival benefit, and DrotAA was withdrawn from clinical use (193, 194).

Several strategies have been explored for targeting NET-mediated immunothrombosis. Peptidylarginine deiminase 4 (PAD4) inhibitors, such as GSK484, suppress NET formation by inhibiting histone citrullination (195). DNase degrades the DNA backbone of the preformed NETs and disrupts their thrombotic scaffolds. Recombinant thrombomodulin neutralizes extracellular histones, thereby reducing endothelial injury and procoagulant activity. Collectively, these approaches offer novel multipronged intervention strategies for sepsis-associated immunothrombosis (140).

As evidence varies substantially across agents, **Table 2** summarizes their clinical and preclinical evidence levels, bleeding risks, and host defense concerns. Despite the identification of numerous candidate targets, most interventions have not consistently improved mortality or disease severity. The biological heterogeneity and mechanistic complexity of sepsis limit the effectiveness of single-pathway interventions. Therefore, future studies should focus on disease stage, mechanistically defined patient subgroups, and the balance between therapeutic benefit, bleeding risk, and preservation of host defense.

**Table 2:** Potential therapeutic agents targeting sepsis-induced immunothrombosis.

Therapeutic strategy/agent	Main target	Mechanism or key findings	Clinical/preclinical evidence level	Bleeding risk/host defense concern	References
Clinically evaluated anticoagulant and antiplatelet strategies					
Low Molecular Weight Heparin (LMWH)​	AT-III	Inhibits factor Xa and thrombin generation; sepsis-specific survival benefit remains unconfirmed.	Established clinical use—widely used for VTE prophylaxis and treatment; sepsis-specific immunothrombosis evidence remains indirect.	Moderate bleeding risk (lower than UFH); renal clearance caution; limited reversibility. Host-defense concern not specifically addressed.	(196)
Recombinant human activated protein C (DrotAA)	Protein C pathway	Anticoagulant and cytoprotective APC pathway. No mortality benefit at 28 or 90 days in septic shock; withdrawn from clinical use as a standard sepsis therapy.	Phase III randomized controlled trials—multicenter studies.	Bleeding concern; nonserious bleeding increased in PROWESS-SHOCK, serious bleeding not significantly different. Host-defense concern not specifically addressed.	(193)
Ticlopidine, Cangrelor, Ticagrelor, etc.	P2Y_12_ receptor	Reduce platelet activation and platelet–leukocyte interactions; no significant improvement in 30-day all-cause mortality.	Observational clinical and human experimental evidence—sepsis cohort and endotoxin-challenge studies.	Bleeding concern; increased gastrointestinal bleeding risk. Host-defense concern not specifically addressed.	(197)
Melatonin	NETosis, endothelial function	Antioxidant, anti-inflammatory, and immunomodulatory effects; reduced thrombosis and mortality in severe COVID-19 patients.	Indirect randomized clinical evidence—single-center, open-label trial in patients with severe COVID-19.	Bleeding or host-defense concern not specifically reported; authors described melatonin as highly safe.	(198)
DrotAA + Heparin	AT-III, protein C pathway	May reduce the risk of ischemic stroke and did not increase 28-day mortality.	Phase IV randomized controlled trial—XPRESS trial.	Any bleeding increased on days 0–6; serious/CNS/fatal bleeding not significantly different. Host-defense concern not specifically addressed.	(199)
Recombinant tissue factor pathway inhibitor(rTFPI)/tifacogin	TF, TF-FVIIa complex, FXa	Inhibits TF-initiated coagulation and reduces thrombin-generation markers, but did not lower 28-day mortality in severe sepsis with high INR.	Phase III randomized controlled trial—OPTIMIST trial.	Increased bleeding events, including serious bleeding adverse events. Host-defense concern not specifically addressed.	(200)
Recombinant Human Soluble Thrombomodulin (rhsTM)	Protein C pathway	Promotes protein C activation; reduced coagulation biomarkers but did not significantly lower 28-day mortality.	Phase III randomized controlled trial—SCARLET trial.	Serious major bleeding events were reported; no anti-drug antibodies were reported.	(201)
AT-III	Thrombin, FXa	Inhibits thrombin and factor Xa; high-dose AT-III did not reduce 28-day mortality; subgroup without heparin showed possible benefit.	Phase III randomized controlled trial—KyberSept trial.	Increased bleeding, especially with concomitant heparin. Host-defense concern not specifically addressed.	(202)
Anti-inflammatory and anticoagulant therapeutic agents under investigation					
C-ST5 peptide	STING pathway	Inhibits platelet STING signaling, NET formation, and septic thrombosis.	Translational preclinical evidence—human cell assays and a murine CLP sepsis model.	Bleeding risk undefined.	(72)
Hemin	Heme oxygenase-1 (HO-1)	Activates HO-1/CO signaling and reduces coagulation and organ injury.	Preclinical *in vivo* evidence—a murine CLP sepsis model.	Bleeding risk not systematically evaluated; prolonged PT/APTT suggests anticoagulant effect.	(203)
Micellar Peptide Inhibitor (MPαC)	Platelet glycoprotein GPIb-IX complex	Blocks GPIb-IX–vWF adhesion and reduces microthrombosis.	Translational preclinical evidence—human platelet–endothelial flow assays and a murine LPS endotoxemia model.	Mild tail bleeding prolongation.	(204)
Reparixin	CXCR1 and CXCR2 receptors	Blocks CXCR1/2 and reduces NETs, fibrin deposition, and organ injury.	Translational preclinical evidence—human plasma assays and murine sepsis models.	Bleeding risk unreported.	(205)
Pinocembrin	Platelet activation	Suppresses platelet activation and septic thrombosis.	Translational preclinical evidence—human platelet assays, murine CLP sepsis and LPS endotoxemia models.	Prolonged tail bleeding time. Host-defense concern not specifically addressed.	(206)
Crocin	PAI-1, t-PA	Restores fibrinolytic balance and reduces renal fibrin deposition.	Preclinical *in vivo* evidence—a rat LPS endotoxemia model.	Bleeding risk unevaluated. Host-defense concern not specifically addressed.	(207)
Parmodulins	Protease-activated receptor 1 (PAR1)	Promote endothelial PAR1 cytoprotective signaling and reduce thromboinflammation.	Translational preclinical evidence—human endothelial assays and a murine LPS endotoxemia model.	No PT/APTT prolongation; low bleeding risk potential. Host-defense concern not specifically assessed.	(208)
HFI-142	Insulin-regulated aminopeptidase (IRAP)	Inhibits IRAP-dependent platelet activation.	Preclinical *in vivo* evidence—murine platelet assays and a murine CLP sepsis model.	Bleeding risk not systematically evaluated; IRAP deficiency prolongs tail bleeding.	(209)
Histidine-rich glycoprotein (HRG)	NETosis	Maintains neutrophil quiescence and reduces NET-associated immunothrombosis.	Translational preclinical evidence—human neutrophil assays and a murine CLP sepsis model.	No direct bleeding evaluation; improved coagulopathy rather than systemic anticoagulation.	(210)
​Stachydrine (STA)	Platelet activation	Suppresses platelet activation, platelet–neutrophil aggregation, and NET formation.	Translational preclinical evidence—human platelet assays and a murine CLP sepsis model.	No significant PT/APTT prolongation; host-defense concern not specifically assessed.	(211)
Embelin	PAI-1	Inhibits PAI-1, enhances fibrinolysis, and reduces septic DIC.	Translational preclinical evidence—human plasma clot-lysis assays and murine thrombosis/septic DIC models.	Prolonged murine tail-bleeding time and hemoglobin loss; host-defense concern not specifically assessed.	(212)
Liraglutide/exenatide	GLP-1R	Inhibits platelet and monocyte activation through GLP-1R signaling.	Translational preclinical evidence—human endothelial cell assays and a murine LPS endotoxemia model.	Bleeding risk unassessed. Host-defense concern not specifically addressed.	(213)
Linagliptin	DPP-4	Reduces endothelial inflammation, TF expression, and microthrombosis.	Translational preclinical evidence—human endothelial cell assays and a murine LPS endotoxemia model.	No direct bleeding evaluation. Host-defense concern not specifically assessed.	(214)
A2 domain peptide of recombinant vWF	Fibrin monomer, vWF-platelet interaction	Blocks flow-dependent platelet adhesion and fibrin-rich microthrombi.	Translational preclinical evidence—human blood-flow assays and murine LPS/DIC models.	No direct bleeding assessment. Host-defense concern not specifically addressed.	(215)
Probenecid nanocrystals (Prob NCs) + Cefotaxime sodium (CTX)	NETosis	Reduces bacterial burden, NET formation, and immunothrombosis.	Preclinical *in vivo* evidence—murine cell assays, murine CLP sepsis and LPS endotoxemia models.	No direct bleeding assessment. Host-defense concern not specifically addressed.	(216)
Anti-CD14 monoclonal antibody	CD14	Suppresses LPS-driven inflammation, coagulation, and organ dysfunction.	Translational preclinical evidence—a nonhuman-primate E. coli sepsis model.	No overt bleeding; important host defense concern (CD14 bacterial recognition/phagocytosis).	(217)
Zixue Powder (ZXP)	STING pathway	Inhibits platelet STING signaling, platelet–neutrophil aggregation, and NET formation.	Preclinical *in vivo* evidence—a murine LPS endotoxemia model.	Bleeding risk not systematically evaluated; component standardization required.	(218)
Cathelicidin-MH (Cath-MH)	Bacterial membranes; coagulation-related proteases; MAPK signaling	Antimicrobial and LPS-neutralizing effects; modulates protease activity and suppresses MAPK signaling, reducing inflammation, organ injury, and mortality	Translational preclinical evidence—human plasma coagulation and *in vitro* protease assays, murine LPS endotoxemia and CLP sepsis models.	Prolonged murine tail-bleeding time; direct antimicrobial activity was demonstrated.	(187)
Cathelicidin-HG (Cath-HG)	Platelet GPVI	Binds GPVI and inhibits Src–Syk–PLCγ2–Akt signaling, attenuating platelet activation, NET formation, organ injury, and mortality	Preclinical *in vivo* evidence—murine platelet assays, CLP sepsis and FeCl_3_-induced carotid thrombosis models.	Bleeding was not directly assessed; organ bacterial burden was reduced in treated septic mice.	(188)
Cathelicidin-LW (Cath-LW)	Bacterial membranes and DNA; coagulation pathways	Combines antimicrobial and LPS-neutralizing activities with anticoagulant effects; reduces inflammation, organ thrombosis, and mortality.	Preclinical *in vivo* evidence—murine LPS endotoxemia, CLP sepsis, and FeCl_3_-induced carotid thrombosis models.	Direct bleeding risk was not evaluated; PT and APTT were prolonged, whereas TT was unchanged.	(186)

Evidence classifications are intended as a descriptive framework for this review and are based on study design, clinical context, and translational proximity. Clinical evidence categories are labeled according to study design and trial phase, including established clinical use, randomized trials, observational or controlled human studies, and indirect randomized evidence from related clinical conditions; established clinical use does not imply proven efficacy against sepsis-specific immunothrombosis. “Translational preclinical evidence” refers to studies combining animal in vivo validation with a substantive human-derived component directly relevant to therapeutic efficacy or mechanism, or studies using clinically relevant large-animal or nonhuman-primate models. “Preclinical in vivo evidence” refers to studies in which the principal efficacy evidence is derived from animal models without a substantive human-derived efficacy or mechanistic component; supporting in vitro assays or ancillary human-cell safety or toxicity testing do not alter this classification.

## 6 Discussion

This review summarizes the current evidence regarding the pathophysiological mechanisms of sepsis-induced immunothrombosis and underscores its role at the interface between inflammation, coagulation, and multiple organ dysfunction. Immunothrombosis is not simply an isolated coagulation abnormality but rather a dynamic thromboinflammatory process that involves coordinated interactions among endothelial cells, platelets, neutrophils, and monocytes/macrophages. Within the microvasculature, endothelial dysfunction, excessive NET formation, complement activation, and the activation of TF-dependent coagulation reinforce each other, thereby sustaining local thromboinflammatory responses and contributing to microvascular dysfunction and organ injury. The organ-focused analysis presented in this review suggests that the heterogeneity of SAOD reflects not only the systemic burden of thromboinflammation but also differences in how individual microvascular beds respond to and translate immunothrombotic stress. This organ architecture-based perspective provides a framework for understanding why a shared systemic immunothrombotic process gives rise to distinct patterns of organ injury during sepsis and may help inform future organ-specific therapeutic approaches. Nevertheless, several limitations of the current evidence should be acknowledged.

First, most of the current evidence is derived from preclinical models, whereas patients with sepsis exhibit substantial biological and clinical heterogeneity. However, the clinical relevance of these findings remains uncertain and requires validation in well-designed prospective studies. The disappointing results of previous coagulation-targeted trials on sepsis have raised questions regarding the causal contribution of microthrombosis to organ injury. Some investigators have proposed that systemic inflammation is the dominant upstream process, with microthrombosis representing a secondary manifestation. This is partly supported by the limited and variable detection of microthrombi in human pathological studies. However, inflammation and thrombosis are mechanistically interdependent; inflammatory signaling promotes a procoagulant microenvironment, whereas NETs, extracellular histones, and activated platelets can further amplify local inflammation. The relative contributions of inflammation and thrombosis are therefore likely to vary according to the disease stage, organ-specific microvascular characteristics, and patient phenotype and remain incompletely defined.

Second, the temporal evolution of immunothrombosis during sepsis remains unclear. Its host-protective and tissue-damaging effects may coexist, rather than occur in strictly separate early and late phases, which makes it difficult to determine the optimal timing and target population for therapeutic interventions. Sepsis-associated immunosuppression may emerge alongside or following hyperinflammation; however, whether and how immunothrombosis contributes to this process remain unclear.

Although several potential targets, including P2Y_12_ signaling, NET formation, and the protein C pathway, have been investigated, their translation into effective therapies remains a challenge. Future interventions are needed to balance the suppression of pathological thrombosis and inflammation with the risk of bleeding and impaired host defense.

Future studies should focus on defining the clinically relevant immunothrombotic phenotypes and identifying biomarkers that support patient stratification. Simultaneously, cell- or pathway-specific interventions should be evaluated in appropriately selected populations and integrated with standard anti-infective and supportive care. These efforts may improve the precision and clinical applicability of immunothrombosis-targeted therapies for sepsis.

## Glossary

SAOD: sepsis-associated organ dysfunction
PAMPs: pathogen-associated molecular patterns
DAMPs: damage-associated molecular patterns
TLRs: Toll-like receptors
PRRs: pattern recognition receptors
TF: tissue factor
TFPI: tissue factor pathway inhibitor
APC: activated protein C
AT: antithrombin
EPCR: endothelial protein C receptor
TM: thrombomodulin
UL-vWF: ultra-large von Willebrand factor
vWF: von Willebrand factor
GAGs: glycosaminoglycans
eGC: endothelial glycocalyx
PAI-1: plasminogen activator inhibitor-1
PF4: platelet factor 4
TXA2: thromboxane A2
NETs: neutrophil extracellular traps
NE: neutrophil elastase
MPO: myeloperoxidase
PS: phosphatidylserine
EVs: extracellular vesicles
ROS: reactive oxygen species
GPVI: glycoprotein VI
CLEC-2: C-type lectin-like receptor 2
STING: stimulator of interferon genes
ALI: acute lung injury
ARDS: acute respiratory distress syndrome
HIF: hypoxia-inducible factor
AKI: acute kidney injury
RVR: renal vascular resistance
iNOS: inducible nitric oxide synthase
SICM: sepsis-induced cardiomyopathy
SIC: sepsis-induced coagulopathy
SILI: sepsis-induced liver injury
LSECs: liver sinusoidal endothelial cells
ET-1: endothelin-1
HSCs: hepatic stellate cells
FDP: fibrin degradation product
SAE: sepsis-associated encephalopathy
BBB: blood-brain barrier
NVU: neurovascular unit
TTP: thrombotic thrombocytopenic purpura
PRES: posterior reversible encephalopathy syndrome
DIC: disseminated intravascular coagulation
HDPs: host defense peptides
AMPs: antimicrobial peptides
APTT: activated partial thromboplastin time
VTE: venous thromboembolism.
